# Physician Awareness of Hereditary Angioedema: A Cross-Sectional Survey with Emphasis on Medication-Related Triggers

**DOI:** 10.3390/jcm15155995

**Published:** 2026-08-01

**Authors:** Nurgul Sevimli, Makbule Seda Bayrak Durmaz, Seda Altıner

**Affiliations:** 1 Department of Immunology and Allergy, Gazi Yasargil Training and Research Hospital, Diyarbakir 21070, Turkey; 2 Department of Immunology and Allergy, Ankara Bilkent City Hospital, Ankara 06800, Turkey; 3 Department of Immunology and Allergy, Ankara University Faculty of Medicine, Ankara 06620, Turkey

**Keywords:** hereditary angioedema, physician awareness, medication-related triggers, angiotensin-converting enzyme inhibitors, clinical education

## Abstract

**Background:** Hereditary angioedema (HAE) is a rare, potentially life-threatening disease in which delayed recognition and inappropriate medication use may result in preventable morbidity and mortality. We aimed to assess physicians’ knowledge regarding HAE-related triggers, clinical features, and management strategies across multiple medical specialties. **Methods:** This single-center, cross-sectional survey was conducted among 350 physicians at a tertiary training and research hospital. A structured electronic questionnaire assessed knowledge of HAE pathophysiology, diagnosis, medication-related triggers, and management. A predefined composite knowledge score (0–14) was calculated. **Results:** Although self-reported familiarity with HAE was high, overall disease-specific knowledge was limited. Awareness of critical medication-related triggers—including angiotensin-converting enzyme inhibitors, dipeptidyl peptidase-4 inhibitors, and estrogen-containing therapies—was low across all specialties, with no significant between-group differences. Substantial knowledge gaps were identified in the recognition of clinical features, diagnostic evaluation, and acute management. In multivariable analysis, prior clinical exposure to HAE patients was the only independent predictor of higher knowledge scores (B = 1.097, *p* = 0.001), whereas specialty group, gender, and years of professional experience were not independently associated with knowledge scores. However, the regression model explained only a small proportion of the variance in knowledge scores. **Conclusions:** Significant gaps in clinically relevant HAE knowledge persist among physicians from multiple medical specialties. Prior clinical exposure was associated with higher knowledge scores, whereas specialty group, gender, and years of professional experience were not independently associated with physician knowledge. Targeted, practice-oriented educational interventions focusing on medication-related triggers and acute management may help bridge these knowledge gaps, complement experiential learning, and ultimately enhance patient safety.

## 1. Introduction

Hereditary angioedema (HAE) is a rare autosomal dominant disorder characterized by recurrent episodes of cutaneous and submucosal edema [1,2]. The pathophysiology centers on dysregulation of the kallikrein–kinin system, leading to excessive bradykinin production and episodic edema involving the skin, gastrointestinal tract, or upper airway [2]. This mechanism sets HAE apart from histaminergic angioedema: attacks are not accompanied by urticaria or pruritus, a key distinguishing feature in clinical practice, and they do not respond to antihistamines, corticosteroids, or adrenaline [2,3].

The most prevalent forms, HAE types I and II, arise from quantitative deficiency or functional impairment of C1 esterase inhibitor (C1-INH), respectively. In both forms, C4 levels are typically low, and diagnosis is established through combined measurement of C4, C1-INH antigenic level, and C1-INH functional activity, as recommended by current international guidelines [2]. HAE with normal C1-INH is a rarer and mechanistically distinct entity encompassing several genetic variants; in these forms, C4 and C1-INH levels are normal and diagnosis relies on clinical criteria and family history, with genetic testing identifying a defined mutation in a subset of cases [2,4]. Current guidelines and most of the available literature on HAE management focus predominantly on types I and II. Accordingly, the present study primarily addresses Hereditary angioedema with C1 esterase inhibitor deficiency (HAE-C1-INH) (types I and II), and the educational implications discussed should be interpreted within this context.

Clinically, cutaneous attacks most often involve the extremities, face, or genitalia and, although disfiguring, are not life-threatening [1,2]. Abdominal attacks cause severe cramping pain and may mimic acute abdomen or familial Mediterranean fever, frequently leading to misdiagnosis and unnecessary surgical interventions [1,2,5,6]. Laryngeal edema, though less common, carries a risk of fatal asphyxia and remains the leading cause of HAE-related mortality [1,2,7].

HAE types I and II are estimated to affect approximately 1 in 50,000 individuals worldwide [2]. Diagnostic delay remains a major challenge; the interval between symptom onset and correct diagnosis can extend from several years to more than two decades across different countries [2,8,9]. In Turkey, the diagnostic interval has been reported to approach 26 years [9], underscoring the magnitude of the problem in certain settings. Misdiagnosis plays a central role in this delay. In a large international registry study, 44% of patients with HAE-C1-INH had received at least one prior misdiagnosis, most commonly allergic angioedema or appendicitis, contributing to significant delays in reaching the correct diagnosis [10].

HAE attacks may occur spontaneously or be triggered by a range of factors. These can be broadly categorized into several groups. Spontaneous attacks may occur without an identifiable precipitant. Other triggers include physical or procedural factors such as trauma, mechanical pressure, and surgical or dental interventions; infectious or inflammatory triggers, particularly upper respiratory infections; psychological stress; hormonal influences; and medication-related factors. Physical stimuli are among the commonly reported triggers [1,2]. Infections may provoke attacks through activation of the contact system, while psychological stress is often reported as a preceding factor [2,3,11]. Hormonal influences, particularly estrogen excess, lower the threshold for attacks and are especially prominent in HAE with normal C1-INH, although estrogen-containing therapies may also exacerbate attacks in HAE-C1-INH [2].

Among medication-related triggers, angiotensin-converting enzyme (ACE) inhibitors impair bradykinin degradation by inhibiting kininase II, while dipeptidyl peptidase-4 (DPP-4) inhibitors reduce its catabolism through an alternative pathway [3,12]. Estrogen-containing therapies, although discussed above in the context of hormonal influences, also represent an important medication-related trigger [2]. These three drug classes, ACE inhibitors, DPP-4 inhibitors, and estrogen-containing therapies, are widely prescribed for comorbid conditions across multiple specialties, and unrecognized exposure in patients with HAE may result in serious yet preventable attacks.

Acute attacks require disease-specific treatment. Current WAO/EAACI guidelines recommend C1-INH concentrate, icatibant, or ecallantide as first-line therapies in confirmed HAE [2]. Established bradykinin-mediated HAE does not respond to adrenaline, antihistamines, or corticosteroids [2,3]. However, in undifferentiated emergency presentations where the diagnosis has not yet been established, conventional allergy treatment may still be administered while the evaluation is ongoing. Once HAE is confirmed or strongly suspected, disease-specific therapy should be initiated without delay [2]. In this context, the possibility of concomitant or alternative histaminergic angioedema should also be considered in the differential diagnosis. For long-term prophylaxis (LTP), options such as C1-INH replacement, lanadelumab, berotralstat, and attenuated androgens are recommended in current guidelines [2].

Although HAE is a rare condition, patients may present to physicians from many different specialties, often before a correct diagnosis is made. Recognizing the disease early is important, as greater awareness among physicians may help shorten diagnostic delay and support timely referral to specialized care. In addition, awareness of modifiable factors, especially medication-related triggers, is essential to prevent avoidable attacks.

However, information on how well physicians outside specialist settings recognize HAE remains limited. Most existing studies have focused on emergency physicians or single specialties [13,14,15], and relatively little attention has been given to medication-related triggers or to factors associated with better knowledge.

In this context, we aimed to evaluate physicians’ awareness of HAE across a broad range of specialties in a Turkish tertiary care center, where diagnostic delay remains considerable. The primary aim was to assess awareness of HAE-triggering medications. Secondary aims were to evaluate knowledge of HAE pathophysiology, clinical presentation, diagnosis, and management; to compare awareness across specialties; and to identify factors associated with higher knowledge levels.

## 2. Materials and Methods

### 2.1. Study Design and Population

This single-center, cross-sectional survey was conducted at Gazi Yaşargil Training and Research Hospital between February and July 2025 following approval by the Institutional Ethics Committee. All physicians actively working at the institution during the study period were invited to participate, irrespective of medical specialty or academic rank.

Approximately 900 physicians were employed at the study institution during the study period, of whom approximately 850 could be contacted via WhatsApp (WhatsApp LLC, Menlo Park, CA, USA; https://www.whatsapp.com, accessed on 7 July 2025) and invited to participate. Participation was voluntary, and 350 physicians completed the questionnaire, corresponding to an approximate response rate of 41.2%.

For the primary analyses, medical specialties were grouped into four clinically meaningful categories based on similarities in prescribing practices, patient populations, and the likelihood of encountering or managing HAE in routine clinical practice. The prescribing/medical specialty group included internal medicine and its subspecialties, cardiology, family medicine and general practitioners, dermatology, and obstetrics and gynecology. The acute/critical care specialty group comprised emergency medicine, anesthesiology, and intensive care. The pediatrics group included pediatrics, pediatric subspecialties, and pediatric emergency medicine. All remaining specialties, including surgical specialties and infectious diseases, were classified as the other specialty group. Subspecialties were grouped under their respective parent specialties.

### 2.2. Data Collection

Data were collected using a structured, self-administered digital survey created via Google Forms (Google LLC, Mountain View, CA, USA; available online: https://www.google.com/forms, accessed on 7 July 2025). The survey link was distributed to eligible physicians through WhatsApp, a communication platform widely used for professional interaction in routine clinical practice. Google Forms was configured to accept only one response per participant to prevent duplicate submissions. All questionnaire items were mandatory; therefore, no partially completed questionnaires or missing data were included in the analysis.

Participation was voluntary, and responses were collected anonymously. No personally identifiable information was obtained, and participation or non-participation had no impact on professional evaluation or institutional responsibilities.

### 2.3. Questionnaire

The questionnaire consisted of 14 items assessing physicians’ knowledge of HAE pathophysiology, clinical presentation, diagnostic evaluation, trigger factors, and management, with particular emphasis on medication-related triggers. It was developed by three allergy and immunology specialists actively involved in the clinical care of patients with HAE. Item selection was based on the 2021 WAO/EAACI guideline and the relevant literature to ensure comprehensive coverage of the principal domains of HAE knowledge [2]. The draft questionnaire underwent three rounds of iterative review and revision among the investigators to improve the clinical relevance, clarity, and wording of the items before administration. Both single-choice and multiple-choice formats were used. The questionnaire was administered in Turkish; an English version is provided in Supplementary Table S1. Formal content validity assessment, face validity testing, cognitive interviewing, construct validity evaluation, factor analysis, and pilot testing were not performed prior to survey administration. In addition, no formal inter-rater assessment was undertaken during questionnaire development.

### 2.4. Assessment of Knowledge and Awareness: Scoring System

A composite HAE knowledge score was constructed to summarize responses across key clinical domains, including pathophysiology, clinical presentation, diagnostic evaluation, trigger recognition, and management. The scoring approach was predefined before data analysis based on current guideline recommendations and the structure of the questionnaire (Supplementary Table S2). Partial-credit scoring was applied only to multi-response items to distinguish partially correct from completely incorrect responses, thereby better capturing different levels of knowledge while avoiding the loss of potentially meaningful information from partially correct responses.

For single-answer items, one point was awarded for a correct response, while incorrect answers and “I don’t know” responses received zero points.

For multi-response items, a structured partial-credit approach was applied to reflect varying levels of knowledge. In the diagnostic evaluation item (Q7), one point was assigned for each correctly identified parameter (serum C4 and C1-INH level and/or function), allowing a maximum of two points.

For the LTP item (Q13), scoring was based on both correct and incorrect selections: two points were awarded if at least one correct option was selected without any incorrect choices; one point was awarded if at least one correct option was selected together with incorrect selections; and zero points were assigned if only incorrect options or “I don’t know” was selected.

The total possible score ranged from 0 to 14, with higher scores indicating greater knowledge of HAE. The internal consistency of the composite knowledge score was evaluated using Cronbach’s alpha to assess the reliability of the questionnaire-derived score. Cronbach’s alpha was 0.731, indicating acceptable internal consistency.

### 2.5. Use of the Knowledge Score in Statistical Analyses

The total HAE knowledge score was treated as a continuous variable and used as the primary outcome measure. The score was compared across demographic and professional characteristics, including gender, years of professional experience, specialty group, and prior clinical exposure to patients with HAE, to explore factors associated with different levels of awareness.

### 2.6. Statistical Analysis

All statistical analyses were performed using IBM SPSS Statistics for Windows, version 27.0 (IBM Corp., Armonk, NY, USA). The distribution of continuous variables was assessed using visual inspection (histograms and probability plots) and formal normality tests (Shapiro–Wilk or Kolmogorov–Smirnov tests, as appropriate). Continuous variables were summarized as mean ± standard deviation (SD) for normally distributed data or as median with interquartile range (IQR) for non-normally distributed data. Categorical variables were presented as frequencies and percentages.

Differences between two independent groups were analyzed using Student’s *t*-test or the Mann–Whitney U test for continuous variables, and the chi-square test or Fisher’s exact test for categorical variables, as appropriate. For comparisons involving more than two specialty groups, differences in total HAE knowledge scores were assessed using the Kruskal–Wallis test due to non-normal distribution of the data.

Associations between continuous variables were evaluated using Spearman’s rank correlation coefficient. To identify factors independently associated with higher HAE knowledge scores, a multivariable linear regression analysis was performed. The total HAE knowledge score was entered as the dependent variable, while specialty group, prior clinical exposure to patients with HAE, years of professional experience, and gender were included as independent variables. Specialty group was modeled using dummy variables, with the “other specialties” group serving as the reference category. Multicollinearity among predictors was assessed using variance inflation factors (VIFs). All statistical tests were two-tailed, and a *p* value < 0.05 was considered statistically significant.

The study protocol was approved by the Local Ethics Committee (Approval No.: 312; 17 January 2025). All participants provided informed consent prior to participation.

## 3. Results

### 3.1. Participant Characteristics

A total of 350 physicians participated in the study. Participant characteristics are summarized in Table 1. Based on the predefined four-category specialty classification, participants were distributed across prescribing/medical specialties, acute/critical care specialties, pediatrics, and other specialties.

### 3.2. Knowledge and Awareness of HAE

Almost all participants reported prior awareness of HAE (99.1%); however, only 49.1% had encountered a patient with HAE in their clinical practice (Table 2).

Regarding disease-related knowledge, 54.3% of participants correctly identified bradykinin as the primary mediator of HAE. While recurrent angioedema attacks (89.1%) and the genetic nature of the disease (87.7%) were widely recognized, only 41.4% correctly identified the absence of urticaria as a characteristic feature of HAE. Knowledge of the diagnostic approach was particularly limited, with only 30.9% (*n* = 108) of physicians correctly identifying serum C4 and C1-inhibitor antigenic level and functional activity as appropriate diagnostic tests.

Awareness of medication-related triggers was also limited. While 45.1% of participants correctly recognized that allergen exposure is not a trigger for HAE attacks, correct identification of established medication-related triggers was considerably lower: 38.6% for ACE inhibitors, 6.9% for DPP-4 inhibitors, and 23.1% for estrogen-containing hormonal therapies. With respect to acute management, only 34.0% of participants correctly identified that adrenaline is not indicated in the treatment of acute HAE attacks (Table 2). Knowledge regarding indications for short-term prophylaxis (STP) was relatively high, with 76.6% of participants providing the correct response, whereas knowledge of LTP was very limited, with only 0.6% of participants providing a fully correct response and 20.3% selecting “I don’t know” (Table 2).

### 3.3. Comparison of Knowledge and Awareness Across Specialty Groups

Awareness of medications known to trigger HAE attacks did not differ significantly across specialty groups. Correct identification of ACE inhibitors as triggering agents ranged from 31.7% to 46.5% across groups, with no statistically significant difference observed (χ^2^ = 5.88, df = 3, *p* = 0.118). Awareness of DPP-4 inhibitors was uniformly low across all specialty groups; correct response rates were 7.9% in prescribing/medical, 7.1% in acute/critical care, and 5.8% in other specialties; the pediatrics group had fewer than five correct respondents, precluding reliable percentage reporting for this subgroup. No statistically significant between-group difference was observed (χ^2^ = 0.44, df = 3, *p* = 0.931).

Similarly, correct recognition of estrogen-containing hormonal therapies as potential triggers was low across all groups (21.4–25.2%), with no significant differences observed (χ^2^ = 0.59, df = 3, *p* = 0.900). Knowledge regarding the non-indication of adrenaline in the treatment of acute HAE attacks also did not differ significantly between specialty groups (χ^2^ = 4.08, df = 3, *p* = 0.253).

Correct identification of bradykinin as the primary mediator of HAE was highest among prescribing/medical (59.8%) and acute/critical care (60.0%) specialties, followed by pediatrics (57.6%), with notably lower recognition in other specialties (44.2%); however, this difference did not reach statistical significance (χ^2^ = 7.60, df = 3, *p* = 0.055).

### 3.4. Factors Associated with Higher Awareness

The overall composite HAE knowledge score was 6.9 ± 3.0 (mean ± SD), with a median (IQR) of 7 (5–9). Analysis of the composite HAE knowledge score revealed variability across physician characteristics. Knowledge scores showed no significant correlation with years of professional experience (Spearman’s ρ = −0.005, *p* = 0.920) and did not differ by gender (Mann–Whitney U = 13,091, *p* = 0.787).

When compared across specialty groups, total HAE knowledge scores showed a non-significant trend toward higher scores among prescribing/medical specialties and pediatrics, with the lowest scores observed in other specialties (Kruskal–Wallis H = 7.457, df = 3, *p* = 0.059).

In contrast, prior clinical exposure to patients with HAE was significantly associated with higher knowledge scores. Physicians who had previously encountered an HAE patient achieved higher median knowledge scores than those without such experience (median [IQR]: 7 [5,6,7,8,9,10] vs. 6 [4,5,6,7,8]; Mann–Whitney U = 11,705.5, *p* < 0.001).

To identify independent predictors of higher awareness, a multivariable linear regression analysis was performed, including specialty group (with other specialties as the reference category), prior clinical exposure to HAE patients, years of professional experience, and gender. The overall model was statistically significant (F = 4.497, *p* = 0.001), although the explained variance was modest (adjusted R^2^ = 0.039). Prior clinical exposure to HAE patients was the only independent predictor of higher knowledge scores (B = 1.097, *p* = 0.001), whereas specialty group, years of professional experience, and gender were not independently associated with the outcome. No evidence of multicollinearity was detected among predictors (all VIF values < 1.1).

## 4. Discussion

In this single-center survey, physicians demonstrated overall limited knowledge of HAE despite a high rate of self-reported familiarity with the condition. Awareness of medications known to trigger HAE attacks, key clinical features, and evidence-based management strategies was suboptimal across all specialty groups, with no statistically significant differences between specialties. Prior clinical exposure to patients with HAE was the only factor independently associated with higher knowledge scores, whereas specialty, gender, and years of experience were not significant predictors. Collectively, these findings suggest that direct clinical experience may contribute to physicians’ awareness of HAE beyond physician characteristics alone.

Analysis of individual knowledge domains revealed an important discrepancy between knowledge of disease mechanisms and their clinical application. More than half of the participants correctly identified bradykinin as the primary mediator of HAE, a proportion somewhat higher than that reported in an earlier emergency physician survey [13]. Awareness of the inherited nature and recurrent course of HAE was also relatively high compared with earlier reports, suggesting that general knowledge of the disease may be improving [13,14]. However, this theoretical understanding did not consistently translate into accurate recognition of clinical manifestations, appropriate diagnostic evaluation, or evidence-based management strategies.

Misconceptions regarding the clinical presentation of HAE were most apparent in relation to urticaria. Fewer than half of the physicians correctly recognized that urticaria is not a typical feature of HAE, a finding consistent with an earlier survey reporting a comparable rate [15]. Because the absence of urticaria is a key distinguishing feature of HAE, failure to recognize this finding may delay consideration of HAE in the differential diagnosis.

Knowledge gaps were also evident in management of acute HAE attacks. Only one-third of participants correctly identified that adrenaline is not indicated for acute HAE episodes, with no significant differences across specialty groups. Similar deficits in acute management, including the misuse of adrenaline, have been reported in previous emergency physician surveys [13,15]. These deficiencies may be further compounded by institutional barriers. An early survey of UK emergency departments found that approximately half lacked a local protocol for C1-INH concentrate administration, despite the drug being available in most units [16]. Current guidelines recommend disease-specific therapies for acute HAE attacks, whereas conventional allergy treatments—including adrenaline, antihistamines, and corticosteroids—are ineffective in patients with confirmed HAE and should not replace targeted treatment [2]. Nevertheless, in undifferentiated emergency presentations where the diagnosis remains uncertain, empirical administration of these agents may be appropriate while the diagnostic evaluation is ongoing. Once HAE is confirmed or strongly suspected, however, continued reliance on conventional allergy therapy alone may delay appropriate management and adversely affect clinical outcomes.

Awareness regarding diagnostic evaluation was notably limited. Definitive diagnosis and screening of HAE-C1-INH- (types I and II) require combined assessment of serum C4 levels and C1-INH antigenic concentration and functional activity [2], yet only 30.9% of physicians in the present study correctly identified this diagnostic approach. Although this proportion was higher than in previous surveys, it remains low overall. Comparably low rates have been documented both nationally and internationally: in an earlier single-center Turkish study, only 7.4% selected the correct combination [14]; in a Turkish survey of internists, only 0.7% identified the correct combination, with 84% having no clear idea of the appropriate diagnostic test [17]; and in a multi-specialty Japanese survey, only 18.9% selected both C4 and C1-INH activity correctly [18]. The persistence of these low rates across settings and over time indicates that laboratory evaluation of HAE remains insufficiently recognized in routine clinical practice.

Awareness of medication-related triggers was limited overall. While 45.1% of participants correctly recognized that allergen exposure is not a trigger for HAE attacks, correct identification of established medication-related triggers was considerably lower: 38.6% for ACE inhibitors, 6.9% for DPP-4 inhibitors, and 23.1% for estrogen-containing hormonal therapies. There were no significant differences between specialty groups. Because medication-related triggers represent a potentially preventable cause of HAE attacks, incomplete recognition of these associations remains clinically relevant [3,11]. The absence of variation across specialties suggests that these gaps are not confined to specific disciplines but may reflect the broader challenge of maintaining practical awareness of rare conditions encountered infrequently in daily practice.

Neither specialty group, years of professional experience, nor gender was independently associated with knowledge scores in the multivariable model. Although a multi-specialty Japanese survey reported significant inter-specialty variation in HAE awareness—in contrast to our null finding for specialty group—that study likewise found no meaningful difference in knowledge across years since graduation [18], echoing the absence of an experience effect in our cohort. Prior clinical exposure to HAE patients was the only independent predictor of higher knowledge scores (B = 1.097, *p* = 0.001), consistent with a similar emergency physician awareness survey in which prior contact with an HAE patient was the only independent predictor of correct answers, whereas academic title and years of professional experience were not [15]. Although the overall model was statistically significant (F = 4.497, *p* = 0.001), it explained only a small proportion of the variance in knowledge scores (adjusted R^2^ = 0.039). Therefore, these findings should be interpreted with appropriate caution. The limited explanatory capacity of the model suggests that physician awareness is likely influenced by additional factors that were not evaluated in the present study. These may include participation in continuing medical education, institutional educational initiatives, previous allergy training, familiarity with current clinical guidelines, and the frequency of clinical exposure to patients presenting with angioedema. Future studies incorporating these variables may provide a more comprehensive understanding of the determinants of physician awareness of HAE.

Overall, these findings point to the need for educational strategies that complement routine medical training by reinforcing practical, experience-oriented knowledge. Importantly, the value of such strategies is not merely theoretical: a recent interventional study among Turkish emergency department physicians demonstrated that a brief, structured educational session significantly improved knowledge of HAE diagnostic criteria and acute management, including a marked increase in the recognition that bradykinin-mediated angioedema does not respond to antihistamine and corticosteroid treatment [19]. Educational approaches that incorporate case-based learning, clinical scenarios, and decision-focused content may help bridge the gap between theoretical understanding and real-world application. Improving physician awareness of medication-related triggers is particularly important for preventing avoidable attacks, while greater familiarity with clinical features and diagnostic approaches may help reduce diagnostic delays. Strengthening the translation of existing knowledge into clinical practice may support earlier recognition, safer prescribing behaviors, and improved outcomes for patients with HAE.

In the present study, awareness regarding STP and LTP strategies for HAE was limited. While most physicians correctly recognized clinical situations in which prophylaxis may be required, such as invasive procedures or surgical interventions, detailed knowledge of the specific pharmacological agents used for STP and LTP was notably low. This pattern suggests that physicians may be familiar with general risk awareness and patient safety principles, yet lack practical familiarity with HAE-specific prophylactic regimens. In routine clinical practice, HAE prophylaxis is usually managed by physicians with dedicated expertise, most commonly allergists or immunologists, or within specialized centers. Consequently, non-specialist physicians may be less familiar with the specific pharmacological details of prophylactic regimens, even when they recognize the relevant clinical indications.

Several limitations of this study warrant consideration. First, the single-center design may limit the external validity and generalizability of the findings. Because the study was conducted at a tertiary referral center, institutional characteristics and local patterns of clinical exposure to HAE may have influenced physician awareness. Consequently, the participating physicians may not be representative of physicians practicing in other healthcare settings, and the findings should be interpreted within the context of the study setting rather than generalized to national or international physician populations. In addition, participation was voluntary, and although the survey was distributed via WhatsApp to approximately 850 physicians who could be contacted, only 350 completed the questionnaire. Therefore, selection bias cannot be excluded, and the participants may not fully represent the target physician population. Consequently, the observed level of physician awareness may have been overestimated. Furthermore, an a priori sample size calculation was not performed because the study aimed to recruit the entire accessible population of eligible physicians at our institution rather than a predetermined sample. This should be considered when interpreting subgroup analyses and the overall findings.

The questionnaire was developed by three allergy and immunology specialists based on the 2021 WAO/EAACI guideline and the relevant literature and underwent three rounds of internal expert review. However, formal validation procedures—including formal content validity assessment, face validity testing, cognitive interviewing, construct validity evaluation, factor analysis, pilot testing, and formal inter-rater assessment—were not performed prior to survey administration. Therefore, the questionnaire and the composite knowledge score should be considered exploratory rather than a formally validated instrument, and the findings should be interpreted accordingly. In addition, several medical specialties were combined into the “Other Specialties” category according to the predefined grouping strategy. Although this approach allowed clinically meaningful comparisons based on similar prescribing practices, patient populations, and clinical roles, the heterogeneous composition of this group may have obscured specialty-specific differences. Finally, the regression model accounted for only a small proportion of the variance in knowledge scores (adjusted R^2^ = 0.039), suggesting that physician awareness is likely influenced by additional factors beyond those evaluated in the present study. As this was a cross-sectional questionnaire-based study, the findings reflect physicians’ knowledge and self-reported responses at a single point in time and may not fully represent real-world clinical decision-making.

Despite these limitations, this study provides a comprehensive evaluation of physicians’ awareness of HAE across multiple medical specialties in a tertiary care setting where patients with HAE are actively managed. The consistently low awareness of medication-related triggers across all specialty groups, regardless of specialty, years of professional experience, or gender suggests that these knowledge gaps are widespread rather than discipline-specific. Limited familiarity with HAE has similarly been reported in single-specialty cohorts, such as a large Brazilian survey of pediatricians [20], and at the international level: in the MENTALIST survey, gaps in non-HAE-expert physician knowledge were ranked among the highest-priority unmet needs by HAE experts across 32 countries [21]. Together, these observations suggest that the deficits identified at our single center reflect a broader, global challenge rather than a purely local phenomenon. Against this background, prior clinical exposure to patients with HAE emerged as the only factor independently associated with higher knowledge scores, suggesting that direct clinical experience may be a key contributor to disease-specific awareness for rare conditions such as HAE.

## 5. Conclusions

This survey demonstrated that, despite high self-reported familiarity with HAE, physicians’ objective knowledge remained limited across multiple clinically important domains, particularly regarding medication-related triggers. Prior clinical exposure to patients with HAE was the only independent factor associated with higher knowledge scores. These findings highlight the need for structured multidisciplinary educational initiatives, including continuing medical education programs, case-based learning, and guideline-oriented training, particularly for physicians involved in diagnosis and prescribing. Such efforts may improve recognition of medication-related triggers, facilitate timely diagnosis, and support the appropriate management of HAE while reducing preventable medication-related attacks. Given the single-center nature of the present study, these findings should be interpreted within the context of the study setting. Future multicenter studies including physicians from diverse healthcare settings are warranted to further characterize physician awareness of HAE and enhance the generalizability of these findings.

## Figures and Tables

**Table 1:** Participant Characteristics.

Variable	*n* (%) or Median (IQR)
Sex (male)	238 (68%)
Age (years)	37 (IQR: 12)
Years in practice	10 (IQR: 11)
Specialty groups according to the predefined four-category classification	
Prescribing/medical specialties	127 (36.3)
Acute/critical care specialties	70 (20.0)
Pediatrics	33 (9.4)
Other specialties	120 (34.3)
Professional title	
Specialist physicians	158 (45.1)
General practitioners	95 (27.1)
Research assistants	63 (18.0)
Assistant professors	4 (1.1)
Associate professors	28 (8.0)
Professors	2 (0.6)

Abbreviations: IQR, interquartile range.

**Table 2:** Overall Knowledge and Awareness of Hereditary Angioedema.

Domain	Correct *n* (%)
General awareness and clinical exposure	
Heard of HAE	347 (99.1)
Encountered HAE patient	172 (49.1)
Knowledge of disease characteristics	
Bradykinin as mediator	190 (54.3)
Recurrent angioedema attacks	312 (89.1)
Genetically inherited disorder	307 (87.7)
Urticaria NOT a feature	145 (41.4)
Diagnostic approach Serum C4 and C1-INH antigenic level and functional activity	108 (30.9)
Trigger factors and medication-related knowledge	
Allergen exposure is NOT a trigger	158 (45.1)
ACE inhibitors as trigger	135 (38.6)
DPP-4 inhibitors as trigger	24 (6.9)
Estrogen as trigger	81 (23.1)
Treatment-related knowledge	
Adrenaline NOT indicated	119 (34.0)
STP indication correct	268 (76.6)
LTP: complete correct response	2 (0.6)
LTP: “I don’t know” response	71 (20.3)

Abbreviations: HAE, hereditary angioedema; C4, complement component 4; C1-INH, C1 esterase inhibitor; ACE, angiotensin-converting enzyme; DPP-4, dipeptidyl peptidase-4; STP, short-term prophylaxis; LTP, long-term prophylaxis.

## Data Availability

The datasets used and/or analysed during the current study are available from the corresponding author on reasonable request.

## Supplementary Materials

The following supporting information can be downloaded at https://www.mdpi.com/article/10.3390/jcm15155995/s1. Table S1: English version of the questionnaire assessing physicians’ knowledge and awareness of hereditary angioedema; Table S2: Scoring System Used for the Hereditary Angioedema Knowledge Assessment.

## Abbreviations

The following abbreviations are used in this manuscript:

ACE inhibitors	Angiotensin-converting enzyme inhibitors
C1-INH	C1 esterase inhibitor
C1-INH-HAE	Hereditary angioedema with C1 esterase inhibitor deficiency
DPP-4 inhibitors	Dipeptidyl peptidase-4 inhibitors
HAE	Hereditary angioedema
LTP	Long-term prophylaxis
STP	Short-term prophylaxis
VIF	Variance inflation factor
