# Supplementary material for: Physician Awareness of Hereditary Angioedema: A Cross-Sectional Survey with Emphasis on Medication-Related Triggers
Source: J Clin Med. 2026 Aug 1;15(15):5995. doi: 10.3390/jcm15155995 (PMC13466214; doi:10.3390/jcm15155995)
Supplement: Supplementary file 1 [file jcm-15-05995-s001.zip › Supplementary Table S1.pdf]

**Supplementary Table S1.** English version of the questionnaire assessing physicians' knowledge and awareness of hereditary angioedema	
Age:	
Gender:	
Years in Career:	
Department:	
Your Title:	☐ General practitioners<br>☐ Research Assistant<br>☐ Specialist physicians<br>☐ Assistant professor<br>☐ Associate professor<br>☐ Professor
**Q1.** Have you ever heard of hereditary angioedema (HAE)?	☐ Yes<br>☐ No
**Q2.** Have you ever encountered a patient with HAE?	☐ Yes<br>☐ No
**Q3.** Which mediator is associated with HAE?	☐ Bradykinin<br>☐ Histamine<br>☐ Leukotriene<br>☐ Prostoglandin
**Q4.** What is the course of HAE disease?	☐ Attacks<br>☐ Persistent<br>☐ I don't know
**Q5.** Which of the following is NOT a symptom of HAE disease?	☐ Urticaria<br>☐ Laryngeal edema<br>☐ Abdominal pain<br>☐ Subcutaneous swelling<br>☐ I don't know
**Q6.** Which of the following statements about HAE is true?	☐ HAE is a genetically transmitted disease<br>☐ HAE is a contagious disease<br>☐ HAE patients always have a family history<br>☐ I don't know
**Q7.** Which tests are used in the diagnosis or screening of HAE (you can mark as many answers as necessary)?	☐ Complement component 3 (C3)<br>☐ Complement component 4 (C4)<br>☐ Total IgE<br>☐ Complement component 9 (C9)<br>☐ Skin Prick Test<br>☐ Specific IgE<br>☐ C1 esterase inhibitor level<br>☐ I don't know
**Q8.** Which of the following is NOT a trigger for HAE disease?	☐ Surgery<br>☐ Dental extraction<br>☐ Infections<br>☐ Trauma<br>☐ Exposure the allergen<br>☐ Stress<br>☐ I don't know
**Q9.** Which medications trigger the attacks of HAE?	☐ ACE inhibitors<br>☐ Calcium Channel Blockers<br>☐ Beta Blockers

	☐ All of them<br>☐ None of them<br>☐ I don't know
**Q10.** Which medications trigger the attacks of HAE?	☐ Metformin<br>☐ GLP-1 analogs<br>☐ SGLT2 inhibitors<br>☐ DPP-4 inhibitors<br>☐ None of them<br>☐ I don't know
**Q11.** Which medications trigger the attacks of HAE?	☐ Antifibrotics<br>☐ Oral Contraseptives<br>☐ Corticosteroids<br>☐ Antibiotics<br>☐ None of them<br>☐ I don't know
**Q12.** Which of the following is NOT used in the treatment of acute attacks of HAE?	☐ Adrenalin<br>☐ C1 esterase inhibitör<br>☐ Fresh frozen plasma<br>☐ Icatibant<br>☐ I don't know
**Q13.** Which of these are used in long-term prophylaxis treatment of HAE? (You can select more than one option)	☐ Systemic corticosteroids<br>☐ Antihistamines<br>☐ C1 esterase inhibitör<br>☐ Danazol<br>☐ Tranexamic acid<br>☐ Icatibant<br>☐ I don't know
**Q14.** Which of the following is a situation where short-term prophylactic treatment of HAE may be necessary?	☐ Dental extraction<br>☐ Delivery<br>☐ Surgery<br>☐ Endoscopy<br>☐ All of them
