# Supplementary material for: Physician Awareness of Hereditary Angioedema: A Cross-Sectional Survey with Emphasis on Medication-Related Triggers
Source: J Clin Med. 2026 Aug 1;15(15):5995. doi: 10.3390/jcm15155995 (PMC13466214; doi:10.3390/jcm15155995)
Supplement: Supplementary file 1 [file jcm-15-05995-s001.zip › Supplementary Table S2.pdf]

Supplementary Table S2. Scoring System Used for the Hereditary Angioedema Knowledge Assessment

Survey Item	Domain	Scoring Rule	Maximum Points
**Q3**	Pathophysiology (mediator)	Correct identification of bradykinin	1
**Q4**	Disease course	Correct identification of recurrent attacks	1
**Q5**	Clinical manifestations	Correct identification that urticaria is NOT a feature	1
**Q6**	Disease characteristics	Correct identification of genetic inheritance	1
**Q7**	Diagnostic evaluation	C4 (1 point) + C1-INH level/function (1 point)	2
**Q8**	Trigger factors	Correct identification of allergen exposure as NOT a trigger	1
**Q9**	Medication triggers (ACE inhibitors)	Correct identification of ACE inhibitors	1
**Q10**	Medication triggers (DPP-4 inhibitors)	Correct identification of DPP-4 inhibitors	1
**Q11**	Medication triggers (hormonal therapy)	Correct identification of oral contraceptives	1
**Q12**	Acute treatment	Correct identification that adrenaline is NOT indicated	1
**Q13**	Long-term prophylaxis	≥1 correct option selected without any incorrect selections (2 points); ≥1 correct option selected with at least one incorrect selection (1 point)	2
**Q14**	Short-term prophylaxis	Correct identification of “all of the above”	1

Total possible score: 14 points

Scoring of Multi-Response Items

Diagnostic evaluation (Q7): One point was awarded for each correctly selected diagnostic parameter (serum C4 and C1 esterase inhibitor level and/or functional activity).

Long-term prophylaxis (Q13): Two points were awarded if at least one correct medication was selected without selecting incorrect options. One point was awarded if at least one correct medication was selected along with incorrect options. No points were awarded if only incorrect options or “I don’t know” was selected.

Abbreviations: C4, complement component 4; C1-INH, C1 esterase inhibitor; ACE, angiotensin-converting enzyme; DPP-4, dipeptidyl peptidase-4.
